# Epidemiologic evaluation of Nhanes for environmental Factors and periodontal disease

**DOI:** 10.1038/s41598-019-44445-3

**Published:** 2019-06-03

**Authors:** P. Emecen-Huja, H-F. Li, J. L. Ebersole, J. Lambert, H. Bush

**Affiliations:** 1 Division of Periodontics, College of Dental Medicine, Medical University of South Carolina, Charleston, South Carolina USA; 2 Providence St. Joseph Health of Oregon, Medical Data and Research Center, Portland, OR USA; 3 School of Dental Medicine, University of Nevada, Las Vegas, Las Vegas, Nevada USA; 4 College of Nursing, University of Cincinnati, Cincinnati, OH USA; 5 Department of Biostatistics, College of Public Health, University of Kentucky, Lexington, KY USA

**Keywords:** Computational biology and bioinformatics, Environmental sciences, Risk factors

## Abstract

Periodontitis is a chronic inflammation that destroys periodontal tissues caused by the accumulation of bacterial biofilms that can be affected by environmental factors. This report describes an association study to evaluate the relationship of environmental factors to the expression of periodontitis using the National Health and Nutrition Examination Study (NHANES) from 1999–2004. A wide range of environmental variables (156) were assessed in patients categorized for periodontitis (n = 8884). Multiple statistical approaches were used to explore this dataset and identify environmental variable patterns that enhanced or lowered the prevalence of periodontitis. Our findings indicate an array of environmental variables were different in periodontitis in smokers, former smokers, or non-smokers, with a subset of specific environmental variables identified in each population subset. Discriminating environmental factors included blood levels of lead, phthalates, selected nutrients, and PCBs. Importantly, these factors were found to be coupled with more classical risk factors (i.e. age, gender, race/ethnicity) to create a model that indicated an increased disease prevalence of 2–4 fold across the sample population. Targeted environmental factors are statistically associated with the prevalence of periodontitis. Existing evidence suggests that these may contribute to altered gene expression and biologic processes that enhance inflammatory tissue destruction.

## Introduction

Despite increasing awareness and improvement in oral health, periodontitis, together with dental caries, remain major health concerns across the lifespan in the United States^1^. Periodontal disease occurs as a result of an interaction between bacterial biofilms and immunoinflammatory responses. It is anticipated that 80% of the risk for periodontal tissue damage is a result of dysregulated host responses against the chronic bacterial insult^2–4^. This interaction can progress to destroy the periodontal tissues and bone, and eventually is the major basis of tooth loss in adults with edentulous individuals having difficulty eating, swallowing, and speaking properly^5–7^. These impaired oral functions can greatly impact individual quality of life, negatively affecting societal and economic opportunities, and continues to expand as a public health concern in aging populations^8^.

Similar to many chronic diseases, it is well documented that periodontal disease is a complex disease with multiple potential contributing factors. These include genetic and epigenetic influences, patient behaviors, medication use, and/or environmental factors, which all together promote periodontal disease initiation and progression^9^. Low socioeconomic status, poor oral hygiene, psychological stress, depression, increased age, Hispanic ethnicity, diet/obesity, and systemic health co-morbidities are well known risk factors that contribute to the prevalence of periodontal diseases^10–12^. However, smoking has been identified as one of the most significant and modifiable risk factor in the pathogenesis of periodontitis and tooth loss^13,14^. Data also support that the number of cigarettes smoked per day is directly related to the prevalence and the severity of the disease^15–17^. Emphasis has been placed on the need for more effective management of these modifiable risk factors to impact this global disease^18^, albeit, non-modifiable factors including age, genetics and the existence of various systemic diseases are clearly more challenging to address across the population^18–21^. In this regard, various studies of this chronic disease have provided some support attributing disease expression and severity to genetic predisposition regulating the characteristics of the host response to the oral microbial challenge. These have included genes controlling the production of inflammatory mediators and tissue and bone regulatory molecules^22–25^ via genetic polymorphisms and more recent reports on epigenetic alterations in the genomes of periodontitis patients^26,27^.

Importantly, studies from other disease models show that various environmental stimuli can contribute to these epigenetic changes and underpin the concept of environment-gene interactions related to disease expression^28^. While rather limited data is available regarding environmental factors in periodontitis^29^, the National Health and Nutrition Examination Survey (NHANES) provides a robust data set regarding measures of 156 environmental factors in blood and urine. This report describes the use of various epidemiologic and statistical tools to conduct an association study with periodontitis in the U.S. adult population.

## Results

The final statistical analysis was completed on 8,884 individuals who were >18 years old and had 16 or more teeth. Males comprised 48.4% of the sample. The majority of subjects were non-smokers (55.9%), and those with smoking experience were evenly distributed between former smokers (22.5%) and current smokers (21.6%). The ethnic distribution of the group was non-Hispanic white (48.5%), non-Hispanic black (18%), Mexican American (25.1%), other Hispanic (4.7%), and other race including multi-racial (3.7%). Approximately 72% of the sample population was older than 30 years of age. (Tables 1 and 2). The weighted prevalence of periodontitis was 8.1% across the entire >18 years of age population. When the periodontitis group defined by NHANES measures was compared to the subset of subjects considered periodontally healthy, individuals with periodontal disease were more likely to be male, older than 30 years of age, Mexican American, non-Hispanic black or Hispanic and current smoker compared to Non-Hispanic white and non-smoker (p < 0.001) (Tables 1 and 3**)**.

**Table 1:** Demographic information by periodontal status.

	N	No Periodontitis	Periodontitis	p-value	Weighted N	Weighted Prevalence of Periodontitis	Weighted p-value
N	8884	7915 (89.1%)	969 (10.9%)	.	137140007	8.1%	.
Male	4297 (48.4%)	3706 (86.2%)	591 (13.8%)	<0.0001	68191901	10.1%	<0.0001
Female	4587 (51.6%)	4209 (91.8%)	378 (8.2%)	.	68948106	6.2%	.
Mexican American	2233 (25.1%)	1913 (85.7%)	320 (14.3%)	<0.0001	11566835	12.3%	<0.0001
Other Hispanic	417 (4.7%)	355 (85.1%)	62 (14.9%)	.	8127478	14.6%	.
Non-Hispanic White	4305 (48.5%)	4030 (93.6%)	275 (6.4%)	.	97058220	5.8%	.
Non-Hispanic Black	1603 (18.0%)	1328 (82.8%)	275 (17.2%)	.	13910439	16.0%	.
Other Race	326 (3.7%)	289 (88.7%)	37 (11.3%)	.	6477034	10.3%	.
Age 18~30	2497 (28.1%)	2390 (95.7%)	107 (4.3%)	<0.0001	36006214	3.1%	<0.0001
Age 31~49	3522 (39.6%)	3068 (87.1%)	454 (12.9%)	.	63781453	9.3%	.
Age 50~64	1667 (18.8%)	1435 (86.1%)	232 (13.9%)	.	26103632	10.9%	.
Age 65+	1198 (13.5%)	1022 (85.3%)	176 (14.7%)	.	11248708	11.6%	.
Age (Mean, StdErr)	43.12 (0.18)	42.47 (0.19)	48.47 (0.49)	<0.0001	41.55 (0.26)	46.51 (0.53)	<0.0001
Non Smoker	4967 (55.9%)	4538 (91.4%)	429 (8.6%)	<0.0001	73898456	5.8%	<0.0001
Current Smoker	1920 (21.6%)	1616 (84.2%)	304 (15.8%)	.	32664766	13.4%	.
Former Smoker	1997 (22.5%)	1761 (88.2%)	236 (11.8%)	.	30576785	8.0%	.
Socio-Eco Status (Mean, StdErr)	2.75 (0.02)	2.82 (0.02)	2.19 (0.05)	<0.0001	3.12 (0.05)	2.50 (0.08)	<0.0001
Total Teeth (Mean, StdErr)	26.19 (0.04)	26.29 (0.04)	25.38 (0.13)	<0.0001	26.26 (0.06)	25.12 (0.17)	<0.0001

**Table 2:** Demographic information by smoking status.

	N	Non Smoker	Current Smoker	Former Smoker	p-value	Weighted N	Weighted Non Smoker	Weighted Current Smoker	Weighted Former Smoker	Weighted p-value
N	8884	4967 (55.9%)	1920 (21.6%)	1997 (22.5%)		137140007	53.9%	23.8%	22.3%	
Male	4297 (48.4%)	2020 (47.0%)	1119 (26.0%)	1158 (26.9%)	<0.0001	68191901	48.1%	26.7%	25.2%	<0.0001
Female	4587 (51.6%)	2947 (64.2%)	801 (17.5%)	839 (18.3%)		68948106	59.6%	20.9%	19.4%	.
Mexican American	2233 (25.1%)	1335 (59.8%)	413 (18.5%)	485 (21.7%)	<0.0001	11566835	58.3%	22.6%	19.2%	<0.0001
Other Hispanic	417 (4.7%)	243 (58.3%)	91 (21.8%)	83 (19.9%)		8127478	55.6%	24.6%	19.7%	
Non-Hispanic White	4305 (48.5%)	2227 (51.7%)	935 (21.7%)	1143 (26.6%)		97058220	51.7%	23.6%	24.7%	
Non-Hispanic Black	1603 (18.0%)	963 (60.1%)	408 (25.5%)	232 (14.5%)		13910439	61.9%	25.8%	12.3%	
Other Race	326 (3.7%)	199 (61.0%)	73 (22.4%)	54 (16.6%)		6477034	59.2%	24.7%	16.1%	.
Age 18~30	2497 (28.1%)	1542 (61.8%)	652 (26.1%)	303 (12.1%)	<0.0001	36006214	57.3%	31.6%	11.1%	<0.0001
Age 31~49	3522 (39.6%)	1932 (54.9%)	923 (26.2%)	667 (18.9%)		63781453	53.7%	25.6%	20.7%	
Age 50~64	1667 (18.8%)	827 (49.6%)	281 (16.9%)	559 (33.5%)		26103632	47.7%	17.2%	35.1%	
Age 65+	1198 (13.5%)	666 (55.6%)	64 (5.3%)	468 (39.1%)		11248708	58.1%	4.5%	37.4%	
Age (Mean, StdErr)	43.12 (0.18)	42.20 (0.24)	37.95 (0.29)	50.38 (0.38)	<0.0001	41.55 (0.26)	41.09 (0.35)	36.82 (0.33)	47.71 (0.41)	<0.0001
Socio-Eco Status (Mean, StdErr)	2.75 (0.02)	2.81 (0.02)	2.26 (0.04)	3.09 (0.04)	<0.0001	3.12 (0.05)	3.21 (0.06)	2.56 (0.07)	3.48 (0.06)	<0.0001
Total Teeth (Mean, StdErr)	26.19 (0.04)	26.55 (0.05)	26.05 (0.08)	25.44 (0.08)	<0.0001	26.26 (0.06)	26.63 (0.06)	25.98 (0.12)	25.67 (0.10)	<0.0001

**Table 3:** Weighted directionality of gender, race and age to prevalence of periodontal disease.

Name	Weighted N	Weighted Percentage of Periodontitis	Name	Weighted N	Weighted Percentage of Periodontitis	Weighted p-value
Male	68191901	10.1%	Female	68948106	6.2%	<0.0001
Mexican American	11566835	12.3%	Other Hispanic	8127478	14.6%	0.3204
Mexican American	11566835	12.3%	NonHispanic White	97058220	5.8%	<0.0001
Mexican American	11566835	12.3%	NonHispanic Black	13910439	16.0%	0.0360
Mexican American	11566835	12.3%	Other race	6477034	10.3%	0.3823
Other Hispanic	8127478	14.6%	NonHispanic White	97058220	5.8%	<0.0001
Other Hispanic	8127478	14.6%	NonHispanic Black	13910439	16.0%	0.5380
Other Hispanic	8127478	14.6%	Other race	6477034	10.3%	0.1266
Non-Hispanic White	97058220	5.8%	NonHispanic Black	13910439	16.0%	<0.0001
Non-Hispanic White	97058220	5.8%	Other race	6477034	10.3%	0.0017
Non-Hispanic Black	13910439	16.0%	Other race	6477034	10.3%	0.0136
Age 18~30	36006214	3.1%	Age 31~49	63781453	9.3%	<0.0001
Age 18~30	36006214	3.1%	Age 50~64	26103632	10.9%	<0.0001
Age 18~30	36006214	3.1%	Age 65+	11248708	11.6%	<0.0001
Age 31~49	63781453	9.3%	Age 50~64	26103632	10.9%	0.0304
Age 31~49	63781453	9.3%	Age 65+	11248708	11.6%	0.0206
Age 50~64	26103632	10.9%	Age 65+	11248708	11.6%	0.5259

Using survey-weighted logistic regression, there were 44 environmental factors (cotinine, 1 dioxin, 4 heavy metals (lead levels in serum and in urine), 8 hydrocarbons, 8 nutrients, 18 PCBs and 3 volatile compounds) that resulted in adjusted odds ratio with p-values < 0.01 for disease versus health in this NHANES cohort (Table 4). When data was further stratified due to smoking status 8 environmental factors (1 heavy metal (lead in serum), and 7 PCBs) in current smokers, 9 factors (acrylamide, 1 heavy metal, 1 nutrient, and 6 PCBs) in former smokers, and 4 factors (2 heavy metals, 1 nutrients, and 1 organophosphate) in non-smokers had FDR values of less than 0.05 (Table 5).

**Table 4:** This table presents a subset of the environmental factors and their association with periodontal disease regardless of the smoking status.

Environmental Factor	Class	Estimated Odds Ratio	95% CI	FDR	N
PCB206 (ng/g)	Pcb	2.65	(1.88, 3.75)	<0.001	1925
PCB172 (ng/g)	Pcb	2.18	(1.61, 2.97)	<0.001	2740
PCB157 (ng/g)	Pcb	2.05	(1.46, 2.87)	0.003	2750
PCB178 (ng/g)	Pcb	2.00	(1.50, 2.66)	0.001	2774
PCB177 (ng/g)	Pcb	1.99	(1.53, 2.60)	<0.001	2740
PCB199 (ng/g)	Pcb	1.96	(1.29, 2.97)	0.023	1967
PCB183 (ng/g)	Pcb	1.84	(1.47, 2.29)	<0.001	2775
PCB194 (ng/g)	Pcb	1.82	(1.23, 2.69)	0.030	1948
PCB196 & 203 (ng/g)	Pcb	1.70	(1.18, 2.43)	0.036	1977
PCB170 (ng/g)	Pcb	1.69	(1.28, 2.23)	0.007	2710
PCB167 (ng/g)	Pcb	1.69	(1.26, 2.27)	0.009	2754
Lead (µg/dL)	heavy metal	1.66	(1.47, 1.87)	<0.001	9208
2-fluorene (ng/L)	hydrocarbon	1.64	(1.38, 1.94)	<0.001	2213
3-fluorene (ng/L)	hydrocarbon	1.63	(1.35, 1.96)	0.001	2201
Benzene (ng/mL)	volatile compound	1.63	(1.27, 2.10)	0.006	1850
Cotinine (ng/mL)	alkaloid	1.56	(1.39, 1.75)	<0.001	9029
PCB153 (ng/g)	Pcb	1.56	(1.18, 2.06)	0.018	2775
Cadmium (µg/L)	heavy metal	1.54	(1.41, 1.68)	<0.001	9208
PCB187 (ng/g)	Pcb	1.53	(1.19, 1.97)	0.014	2775
PCB156 (ng/g)	Pcb	1.52	(1.13, 2.05)	0.038	2758
Toluene (ng/mL)	volatile compound	1.51	(1.20, 1.88)	0.009	1909
PCB146 (ng/g)	Pcb	1.51	(1.18, 1.94)	0.014	2771
PCB105 (ng/g)	Pcb	1.49	(1.22, 1.82)	0.004	2764
Cadmium, urine (ng/mL)	heavy metal	1.47	(1.16, 1.85)	0.016	2993
1-pyrene (ng/L)	hydrocarbon	1.46	(1.19, 1.79)	0.011	2208
1-napthol (ng/L)	hydrocarbon	1.43	(1.22, 1.69)	0.004	2245
PCB66 (ng/g)	Pcb	1.43	(1.18, 1.72)	0.007	2757
2-napthol (ng/L)	hydrocarbon	1.42	(1.18, 1.70)	0.009	2237
2,3,7,8-tcdd (fg/g)	dioxins	1.41	(1.19, 1.68)	0.005	2433
2-phenanthrene (ng/L)	hydrocarbon	1.41	(1.14, 1.74)	0.023	2203
1-phenanthrene (ng/L)	hydrocarbon	1.38	(1.17, 1.63)	0.009	2204
3-phenanthrene (ng/L)	hydrocarbon	1.36	(1.15, 1.61)	0.010	2173
Styrene (ng/mL)	volatile compound	1.36	(1.10, 1.69)	0.036	1820
Antimony, urine (ng/mL)	heavy metal	1.28	(1.12, 1.45)	0.006	2999
Retinyl stearate (µg/dL)	nutrient	1.19	(1.08, 1.32)	0.011	7925
α-tocopherol (µg/dL)	nutrient	1.16	(1.05, 1.27)	0.028	8717
Folate, RBC (ng/mL RBC)	nutrient	0.85	(0.76, 0.94)	0.021	9169
Vitamin D (ng/mL)	nutrient	0.83	(0.73, 0.93)	0.023	6404
trans-β-carotene (µg/dL)	nutrient	0.81	(0.70, 0.93)	0.036	6422
Folate, serum (ng/mL)	nutrient	0.80	(0.71, 0.90)	0.009	9134
β-cryptoxanthin (µg/dL)	nutrient	0.80	(0.71, 0.91)	0.013	6401
α-Carotene (µg/dL)	nutrient	0.80	(0.68, 0.93)	0.039	6420
Lead, urine (ng/mL)	Heavy Metal	1.29	(1.07, 1.55)	0.041	3075
PCB138 & 158 (ng/g)	Pcb	1.43	(1.1, 1.87)	0.046	2770

The Odds Ratio estimates, Standard Errors, 95% CI, and FDRs are calculated based on the survey weighted logistic regression with dichotomous periodontitis status as the outcome adjusting for age, gender, ethnicity, socioeconomic status and number of teeth. All environmental variables were log-transformed (natural) and standardized, and the estimates should be interpreted on the same scale. Due to missingness in the data, the sample sizes were not the same for most of these analyses. Exclusion of smoking status resulted in higher N numbers for folate, cadmium, cotinine and lead.

**Table 5:** Environmental variables and the parameter estimates from survey-weighted logistic regressions stratified by smoking groups.

		Current Smokers				Former Smokers				Non-Smokers			
		Est OR	95% CI	FDR	N	Est OR	95% CI	FDR	N	Est OR	95% CI	FDR	N
Lead	Heavy metal	1.54	(1.28, 1.87)	0.006	1931	1.57	(1.27, 1.94)	0.008	2114	1.39	(1.18, 1.65)	0.019	4428
PCB105 (ng/g)	Pcb	1.68	(1.28, 2.2)	0.018	588	1.89	(1.39, 2.57)	0.008	638	1.41	(1.11, 1.78)	0.098	1316
PCB172 (ng/g)	Pcb	2.59	(1.6, 4.21)	0.017	587	4.42	(2.12, 9.23)	0.010	632	1.69	(1.19, 2.4)	0.098	1311
PCB177 (ng/g)	Pcb	2.18	(1.42, 3.33)	0.022	585	3.12	(1.69, 5.75)	0.016	627	1.75	(1.24, 2.46)	0.090	1307
PCB178 (ng/g)	Pcb	2.65	(1.65, 4.25)	0.013	591	2.78	(1.59, 4.86)	0.017	637	1.58	(1.15, 2.16)	0.098	1323
PCB206 (ng/g)	Pcb	3.96	(1.96, 8.01)	0.022	434	5.29	(1.68, 16.65)	0.122	439	1.96	(1.3, 2.94)	0.092	912
PCB183 (ng/g)	Pcb	2.23	(1.54, 3.22)	0.008	593	2.03	(1.23, 3.33)	0.120	640	1.53	(1.12, 2.09)	0.119	1321
PCB170 (ng/g)	Pcb	1.98	(1.35, 2.92)	0.026	585	1.36	(0.67, 2.76)	0.756	625	1.2	(0.84, 1.73)	0.575	1287
PCB157 (ng/g)	Pcb	2.3	(1.31, 4.04)	0.069	584	4.66	(2.14, 10.13)	0.011	633	1.77	(1.23, 2.55)	0.092	1313
PCB66 (ng/g)	Pcb	1.63	(1.2, 2.21)	0.055	584	1.83	(1.4, 2.38)	0.004	635	1.24	(0.95, 1.62)	0.334	1319
Acrylamide (pmoL/G Hb)	Acrylamide	1.46	(1.06, 2.03)	0.237	622	0.3	(0.2, 0.45)	0.013	643	0.77	(0.54, 1.1)	0.450	1301
Vitamin D (ng/mL)	Nutrient	1.15	(0.91, 1.45)	0.584	1393	0.61	(0.5, 0.74)	0.004	1485	0.76	(0.67, 0.87)	0.019	3044
Antimony, urine (ng/mL)	Heavy metal	0.87	(0.65, 1.16)	0.672	634	1.45	(1.05, 1.98)	0.186	705	1.51	(1.27, 1.8)	0.004	1407
Diethylphosphate (µg/L)	Organo-phosphates	0.76	(0.62, 0.94)	0.109	593	0.8	(0.57, 1.11)	0.640	618	1.57	(1.24, 1.99)	0.023	1342
Mono-n-methyl phthalate	Phthalate	1.47	(1.13, 1.92)	0.081	496	0.71	(0.44, 1.15)	0.640	513	0.99	(0.75, 1.31)	0.972	1073
Cadmium (µg/L)	Heavy metal	1.32	(1.09, 1.58)	0.069	1931	1.32	(0.96, 1.83)	0.501	2114	1.26	(1.03, 1.55)	0.204	4428
1,2,3,7,8-pncdd (fg/g)	Dioxins	1.66	(1.16, 2.37)	0.080	533	1.09	(0.71, 1.67)	0.967	596	1.01	(0.76, 1.34)	0.972	1226
2,3,7,8-tcdd (fg/g)	Dioxins	1.81	(1.21, 2.7)	0.069	535	1.63	(1.13, 2.35)	0.136	590	1.22	(0.99, 1.49)	0.290	1213
PCB146 (ng/g)	Pcb	1.71	(1.23, 2.4)	0.055	591	1.87	(1.13, 3.09)	0.166	637	1.23	(0.84, 1.79)	0.558	1321
PCB167 (ng/g)	Pcb	1.77	(1.23, 2.57)	0.065	583	2.93	(1.29, 6.7)	0.151	635	1.61	(1.11, 2.34)	0.143	1315
PCB187 (ng/g)	Pcb	1.75	(1.19, 2.56)	0.075	592	1.47	(0.76, 2.82)	0.647	641	1.15	(0.92, 1.45)	0.495	1049
Retinyl palmitate (µg/dL)	Nutrient	1.35	(1.09, 1.67)	0.081	1797	0.98	(0.77, 1.24)	0.976	1938	1.03	(0.88, 1.22)	0.889	4079
Retinyl stearate (µg/dL)	Nutrient	1.32	(1.09, 1.59)	0.069	1711	1.28	(1.05, 1.57)	0.166	1831	1.07	(0.92, 1.25)	0.628	377
cis-b-carotene (µg/dL)	Nutrient	0.95	(0.81, 1.13)	0.853	1395	1	(0.73, 1.38)	0.992	1481	0.78	(0.67, 0.92)	0.098	3055
1,2,3,4,6,7,8,9-ocdd (fg/g)	Dioxins	1.22	(0.89, 1.67)	0.548	525	1.12	(0.55, 2.27)	0.967	579	1.46	(1.12, 1.91)	0.099	1189

This table presents a subset of the environmental factors and their association with periodontal disease. Environmental variables included are those with False Discovery Rate of <0.05 in at least one of the smoking groups highlighted in yellow and an FDR <0.1 highlighted in orange. The Odds Ratio estimates, Standard Errors, 95% CI, and FDR are calculated based on the survey weighted logistic regression with dichotomous periodontitis status as the outcome adjusting for age, gender, ethnicity, socioeconomic status and number of teeth. All environmental variables were log-transformed (natural) and standardized, and the estimates should be interpreted on the same scale. Due to missingness in the data, the sample sizes were not the same for most of these analyses.

In regression analyses considering each environmental factor separately, blood lead levels were consistently identified as a factor in both the overall and stratified analyses ([a]OR = 1.54, 95% CI: (1.28,1.87) for current smokers; [a]OR = 1.39, 95% CI: (1.18,1.65) for non-smokers; [a]OR = 1.57, 95% CI: (1.27,1.94) for former smokers) (Table 5). Among the 17 polychlorinated biphenyls (PCBs) found to be associated with periodontitis in the overall sample, 6 (i.e. PCB105, PCB157, PCB172, PCB177, PCB178, and PCB206) were also found to have estimated adjusted odds ratios ranging from 1.41 to 5.29. In addition, across these environmental variables, the adjusted OR estimates were lower in non-smokers compared to current and former smokers. The smoking population also demonstrated additional factors, including 6 PCBs (PCB66, PCB146, PCB167, PCD170, PCB183, PCB187) with adjusted OR estimates from 1.63–2.23. It might be expected that the relationship between the array of PCBs and periodontitis risk would be highly correlated. Of the 8 coplanar PCBs (28, 66, 74, 105, 118, 156, 157, 167), we noted significant correlations among these ranging from 50–100% of the other coplanar agents. Similarly 70–90% of the 16 non-coplanar PCBs were significantly correlated within this category, while only 10 showed correlations with the coplanar congeners. Thus, there was some measure of independence in these relationships that would enable future more granular description of specific PCBs periodontitis risk and/or severity. Dioxins (PNCDD, TCDD) showed adjusted OR estimates of 1.66 and 1.81, and blood nutrients retinyl stearate and retinyl palmitate exhibited adjusted OR estimates from 1.32–1.35. In contrast, blood nutrients such as Vitamin D and cis-ß-carotene were estimated to be protective for periodontitis. Higher levels of Vitamin D estimated to decrease the odds of periodontitis by 39% and 24% in former and non-smoker groups, respectively ([a]OR = 0.61, 95% CI: (0.50, 0.74) for former smokers; [a]OR = 0.76, 95% CI: (0.67, 0.87) for non-smokers), and cis-ß-carotene was estimated to decreasing the odds of periodontitis by 22% in non-smokers ([a]OR = 0.78, 95% CI: (0.67, 0.92)) (Table 5).

**Figure 1:**
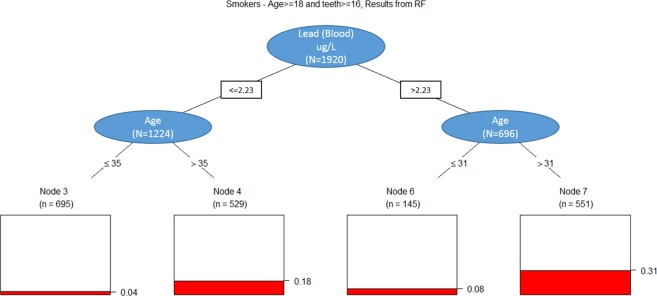
Classification and Regression Tree (CART) analyses on smoker population.

**Figure 2:**
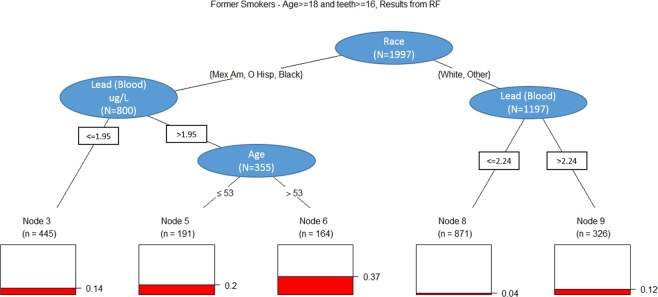
Classification and Regression Tree (CART) analyses on former smoker population.

**Figure 3:**
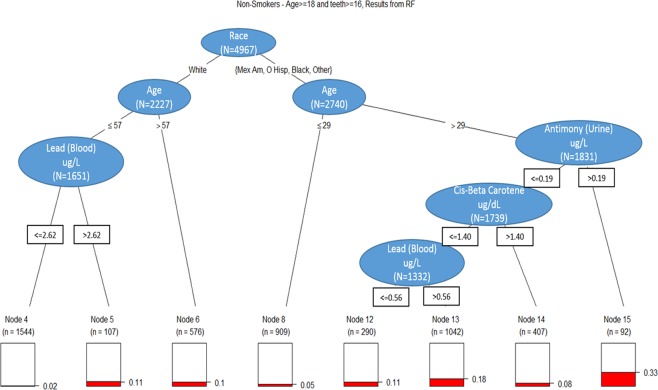
Classification and Regression Tree (CART) analyses on non-smoker population.

We subsequently employed Random Forests (RF) and Classification and Regression Tree (CART) analyses to identify and visualize relationships of critical demographic and environmental factors. Based upon the variables, which had high importance in the RF for each smoking status, a CART was performed separately for each of the smoking, former smoking, and non-smoking subsets. CART analysis on the smoking population, presents elevated blood lead levels as an initial discriminator, with age >35 yrs. stratifying patients with an approximate 4-fold prevalence of periodontitis. (Fig. 1) CART analysis on former smoker population, visualizes the factors classifying the disease risk in former smokers. In this case, race/ethnicity remained a critical factor. Those who reported race/ethnicity other than non-Hispanic white demonstrated increased disease prevalence; elevated blood lead levels and age >53, had an increased periodontitis prevalence of 37%. Within the subset of non-Hispanic white subjects and other race including multi-racial, a prevalence rate of 12% was observed in those with elevated blood lead levels. (Fig. 2**)** For non-smokers, which comprised 56% of the total population, multiple variables were identified to have relationships with periodontal disease status. Race-ethnicity and age were important distinguishing factors. The prevalence was low across those reporting a non-Hispanic white race, but even in this group subjects >44 years demonstrated an increased prevalence. The prevalence was further modified by elevated urine antimony that increased the observed prevalence of periodontitis to 33% from as low as 8% in the low urine antimony and high cis-ß-carotene group. For those with low levels of cis-ß-carotene, higher blood lead levels showed a higher prevalence of periodontitis of 18% compared to 11% for the lower blood lead group. (Fig. 3**)**. This data analysis exercise represents an approach consistent with the current trend in precision health, in that identification of risk and use of model for early prediction of disease initiation/progression will be critical for future improvement of oral health and it impacts on systemic health in the population.

## Discussion

The current paradigm of periodontitis is that it represents a dysregulation of the host response to a dysbiotic microbiome that occurs in a large portion of the global population. Substantial work is being conducted via the Human Microbiome Project^30^ to discern not only the characteristics of the alterations in the disease microbiome, but also interrogating complex metagenomic datasets to assess functional changes in the microbial ecology associated with health and disease^31^. Additionally, a complementary research direction is attempting to document the role of individual genetic variation across the population that contributes to disease expression and severity^19^. These studies have employed SNP analysis of specific targeted genes^19,22,32^, Genome-Wide Association Studies (GWAS)^24,33^ and epigenetic analyses^26,34,35^ to help elucidate the complex of factors that interact to create a disease susceptible host. This report describes an additional consideration in disease expression focused on the larger environmental variation to which individual members and subgroups of the U.S. population are exposed (*i.e*. exposome) as a potential direct contributor to the microbial dysbiosis^36^ and/or a modifier of host responses through altered molecular pathways or modulation of genetic control of the disease^27,37^. The findings identified more classical factors (*i.e*. age, gender, race/ethnicity) in the disease model, but for the first time integrated a subset of environmental factors, both toxins and nutrients, that appear to substantially modify the prevalence of periodontitis in the population. The identification of the association of environmental toxins including lead, hydrocarbons, polychlorinated biphenyls, and nutrients such as retinyl stearate in models described an increase in the prevalence of disease. Thus, the findings support the potential for a role of these factors in modifying the challenge (i.e. bacterial biofilms) and/or host responses with a loss of homeostasis and tissue destruction.

The results demonstrated altered levels of various heavy metals, including lead, cadmium and antimony in periodontitis patients. A range of literature has shown the toxic properties of systemic elevations in heavy metals from environmental sources, including lead^38,39^. In particular, this toxin has been linked to substantial neurotoxicity and negative developmental processes in children^40,41^. This study identified, using CART analysis, an estimated threshold of >2.0 µg/dL that discriminated periodontitis from health in the adult population. While this level does not indicate the actual blood lead level across the periodontitis group, since CART attempts to fit the discrimination profile in the context of multiple variables, it was clear that in all subsets of smokers, former smokers and non-smokers that lead levels were elevated in periodontitis patients. An earlier evaluation of data from NHANES III (1988–94) demonstrated a significantly increased OR for periodontitis in both men and women with increased blood lead levels^29^. Reports examining various iterations of the Korean NHANES (KHANES) study demonstrated elevated lead, cadmium, or mercury in subjects with periodontitis, particularly related to smoking and in some instances gender associated similar to our data from NHANES^42–45^. An additional study reported that chronic occupational exposure of workers to lead resulted in significant changes in oral health and correlated with increasing blood lead levels^46^. Terrizzi *et al*., have reported that elevated lead levels under hypoxia induces alveolar bone resorption and periodontitis^47^. More recently they demonstrated that iNOS and PGE_2_ levels are altered by lead and hypoxia as inflammatory responses that would contribute to damage of the periodontium^48^. Furthermore, the lead levels investigated in these previous studies generally targeted levels that have been shown in blood to have substantial neurotoxicity (≥10 µg/dL), although levels of >5 µg/dL are considered deleterious^49^. Further studies will be required to identify the relationship of blood lead levels to severity of the disease, age of onset, and response to therapy, as well as biologic studies determining the impact of these altered levels of lead on host responses, and even the microbial ecology related to the disease process.

Polychlorinated biphenyls (PCBs) were once widely deployed as dielectric and coolant fluids in electrical apparatus, carbonless copy paper, and in heat transfer fluids since they do not easily degrade. PCBs’ environmental toxicity and classification as a persistent organic pollutant resulted their production and use of them being banned by the United States Congress in 1979. Coplanar PCBs, *e.g*. dioxin-like PCBs, since their structure is similar to dioxins, allows them to act as agonists of the aryl hydrocarbon receptor (AhR). They are considered as contributors to overall dioxin toxicity within the environment. The toxicity of PCBs varies considerably among various chemical structural iterations with the coplanar PCBs representing 12/209 possible PCB molecules (*i.e*. PCB 77, 81, 114, 118, 123, 126, 156, 157, 167, 169, 189) generally considered among the most toxic congeners with the majority of differences occurring in smokers and former smokers. Interestingly, the overall group of toxins included PCB105, PCB146, PCB172, PCB177, PCB178, PCB183, and PCB206, which are all members of the non-coplanar group of PCBs appeared to show the most frequent association with periodontitis. Elevated levels of non-coplanar PCBs, including PCB153, PCB170, PCB180 and PCB187 were detected in the blood of Canadian First Nations communities and were associated with elevated levels of an array of immune activation markers including IFNγ, IL-1ß, IL-8, IL-17A and TNFα^50^. Much of the molecular aspects of PCBs and host responses have focused on the coplanar, dioxin like congeners. The current study identified an array of PCBs that were increased across the periodontitis population. While some representative true dioxin molecules had increased OR for periodontitis these only were noted in smokers. No other reports are available identifying PCB levels and periodontitis in humans or animal models, nor focusing on biologic alterations in cells related to periodontal health and disease, thus, this family of exposome factors could present an important area for further investigation of disease variation and personalized documentation of disease features within the population. Finally, a single recent report demonstrated that PCB126 appeared to exacerbate periodontal disease in a susceptible species of mink^51^.

An interesting finding was the dichotomy between the effects of selected specific nutrients on the expression of periodontitis. Both carotenoids and Vitamin D levels had adjusted Odds Ratios which suggested that they were protecting against periodontitis. Carotenoids are organic pigments found in plants and some photosynthetic microorganisms and carotenoids from human diets are stored in the fatty tissues. There are over 600 known carotenoids classified as xanthophylls (β-cryptoxanthin, lutein, and zeaxanthin; non-vitamin A carotenoids) and carotenes (α-carotene, β-carotene, and lycopene). Generally, the health benefits of carotenoids are thought to be due to their role as antioxidants with dietary carotenoids proposed to interact with endogenous antioxidant enzymes to positively affect immunity^52^. Thus, various reports have shown that elevations in acute phase proteins are accompanied by low vitamin A levels^53^ and that carotenoids significantly reduced proinflammatory cytokines, CRP, and other markers of inflammation in multiple tissues^54^. A study of inflammation in 60–70 year old men demonstrated an inverse relationship between elevated carotenoids and serum CRP levels^55^. Moreover, low blood levels of various carotenoids have been associated with an increased prevalence of periodontitis in 60–70 year old men^56^ and carotenoid levels were related to positive outcomes of scaling and root planing with the relationship limited to non-smokers^57^. Thus, our data from a large population cohort is consistent with these findings and the support that increased availability of carotenoids appears to provide some level of protection from periodontitis.

Vitamin D has received an increasingly detailed examination regarding its potential influence in periodontitis. Various reports have linked decreased serum or saliva vitamin D levels with tooth loss and periodontitis^58–62^ including in smokers^63^, albeit not all studies are supportive since this was not observed in postmenopausal women^64^. Additionally, a gene polymorphism for vitamin D binding protein increases the risk for periodontitis^65^ that appears exacerbated in smokers^25^. Our analysis of this nutrient was based upon examination of NHANES data, and demonstrated an estimated protective feature of this serum nutrient in periodontitis, specifically in non-smokers and former smokers. This type of finding is consistent with additional associational data from NHANES related to risk of cardiometabolic disease^66^, asthma^67^, and coronary heart disease and all-cause mortality^68^. Interestingly, a single recent report describes the interaction of an environmental exposure to phthalates may decrease blood levels of vitamin D^69^, an observation consistent with our results identifying “competing” impact of environmental toxins and nutrients on periodontitis as the clinical outcome.

In contrast, elevated levels of retinyl stearate and retinyl palmitate were each estimated to enhance the risk for periodontitis particularly in smokers. The retinoids comprise a class of compounds related to Vitamin A. These compounds have been used to regulate epithelial cell growth, as well as playing a role in vision, regulation of cell proliferation and differentiation, growth of bone tissue, immune functions, and even activation of tumor suppressor genes^70^. Our data demonstrated an increased OR for blood levels of retinyl stearate and retinyl palmitate in periodontitis. In serum, 56% of retinyl esters are retinyl stearate, 33% retinyl palmitate, and 5% retinyl oleate. Retinyl esters in humans are derived from animal sources and are hydrolyzed in the intestinal lumen to form retinol and fatty acids, such as retinyl palmitate or stearate. Enzymes in the intestinal lumen that hydrolyze dietary retinyl esters include cholesterol esterase from the pancreas and a retinyl ester hydrolase intrinsic to cells of the small intestine, which primarily acts on long-chain fatty acids, such as palmitate or stearate^70^. A single study has been reported regarding these compounds and periodontitis. Wang *et al*.^71^ demonstrated that all-trans retinoic acid administration modulated the Th17/Treg balance and can modulate the expression of periodontitis in a murine model of *P. gingivalis* infection and provided protection against periodontitis with increased Treg activation and decreased Th17 functions. However, our data specifically related to endogenous levels of a specific retinoid, retinyl stearate, suggested an increased risk for periodontitis. This may relate to the more individualized functions of the various members of this family of dietary nutrients, and may highlight some unique features of the diet or intrinsic variation in the hydrolytic enzymes across the population that may link retinyl stearate and disease. Clearly additional studies will need to be conducted examining in more detail the clinical relationship with this compound, as well as its potential role in affecting an array of inflammatory responses that would be related to periodontitis.

This report describes an associational study of a large U.S. population sampled cross-sectionally during a 5 year interval via the NHANES project and demonstrated statistical associations of a subset of environmental challenges to the expression of periodontitis. A clear limitation in the approach is that the findings do not deliver any cause and effect relationship, and are affected by the lack of detailed clinical evaluation of periodontitis that is generally accepted within the field. However, the model developed identified an interaction of these exposome factors and more classical risk factors of age, gender, and race/ethnicity, thus providing some confidence that the findings are providing additional clues into population variation in disease expression. The model will also enable future testing with additional NHANES datasets, as well as the environmental features and categorization of disease. The individual exposome components that were identified can be further evaluated in more detailed clinical studies, and by implementing basic biologic studies of the host cells and microbiome components associated with health and disease to delineate modes of actions of these environmental factors that could contribute to the disease processes.

## Materials and Methods

### Population data

The NHANES is a complex, multistage probability sample of non-institutionalized U.S. civilians and subsequently organized into 6 unique datasets derived from 2-year cycle population sampling (Centers for Disease Control and Prevention; National Center for Health Statistics). Each 2-year survey cycle examines a representative U.S sample of approximately 10,000 persons and collects health-related data. Full descriptions of the sample design for these NHANES datasets are publically available (https://www.cdc.gov/nchs/nhanes/). These surveys, using the same methods, assessed the health status of a nationally representative sample of the civilian non-institutionalized US population, selected through a stratified multistage probability sampling design. In this study, periodontal examination data from three NHANES cohorts, 1999–2000, 2001–2002, 2003–2004, were extracted and combined to comprise the study population. NHANES 1999–2000 (N = 9956), 2001–2002 (N = 10,477) and 2003–2004 (N = 9643) enlisted persons 1 mo of age or older (https://wwwn.cdc.gov/nchs/nhanes/Default.aspx). The analysis for this study included only the records of participants who were equal to and older than 18 years of age and had 16 or more teeth, which resulted 3,745 of participants in the first cohort (1999–2000), 4,258 participants in the second cohort (2001–2002), and 3,834 participants in the third cohort (2003–2004) Thus, the combined sample was 11,387. Those with missing smoking status and periodontal parameters were excluded leaving a final analytical sample of 8,884 participants.

These data have been merged and processed and can be found at https://github.com/joshuawlambert/PinarEtal2018/raw/master/data.zip. A unique identifier, SEQN https://wwwn.cdc.gov/Nchs/Nhanes/1999–2000/DEMO.htm#SEQN for the NHANES participant from our years of study (1999–2004) is included in these data.

### Demographics

The demographic variables considered in this study included age, gender, race, socio-economic status, smoking status, and number of teeth. Racial-ethnic groups were summarized into five categories: Mexican American, Other Hispanic, Non-Hispanic White, Non-Hispanic Black, and Other Race. Socio-economic status, estimated using the poverty income ratio, was computed as the ratio of family/individual income to the appropriate federal poverty threshold. Smoking status, current smoker, former smoker, non-smoker, was derived from the two self-reported questions. Participants reported having historically smoked more than 100 cigarettes, but currently not smoking were defined as former smokers. Non-smokers were defined as reporting never smoking.

### Clinical parameters

Periodontitis was defined as a minimum of 2 or more sites with clinical attachment loss (CAL) ≥3 mm and a periodontal pocket ≥4 mm as described by Eke *et al*.^72^. NHANES (1999–2004) used the partial-mouth periodontal examination (PMPE) protocol to sample teeth and sites. The PMPE protocols randomly selected two quadrants of the mouth and specified 2 to 3 sites per tooth for measurement of pocket depth, attachment loss, and bleed on probing. In 1999–2000, two sites per tooth (mid-facial and mesio-facial) were measured, while three sites per tooth (mid-facial, mesio-facial and distal) were measured in 2001–2002 and 2003–2004. Dentists trained in the survey examination protocol conducted the periodontal examinations collecting probing depth and attachment loss and bleeding on probing measurements^73–75^.

### Environmental variables

The environmental factors were categorized into 15 classes based on NHANES categorization. Environmental variables measured in at least one of the three data cohorts (i.e. 1999–2004) were included in the study. A total of 156 environmental factors were measured in the NHANES data using blood and urine samples. These included chemical toxicants, pollutants, allergens, bacterial/viral organisms and nutrients. Environmental factors with laboratory measurements that had greater than 10% of the observations below a detection limit threshold defined by NHANES were omitted from analysis. The laboratory measurements using mass spectrometry and absorption spectroscopy demonstrated that the majority of the variables were detected in small ranges and were skewed and thus all 156 environmental variables were log-transformed (natural), standardized, and referred to as “processed”.

### Statistical approaches

Survey-weighted logistic regressions were performed for each of the processed environmental factors, adjusting for age, gender, ethnicity, socio-economic status, smoking status and number of teeth. The R package “survey” was used in R (Version 3.1.2) for the survey-weighted logistic regression. Weights were constructed in SAS (Version 9.4) using a 6 year weighting design from the NHANES variable WTMEC2YR73 (http://www.cdc.gov/nchs/tutorials/Nhanes/SurveyDesign/Weighting/Task2.htm). Survey weighted logistic regression seeks to minimize bias by weighting the samples to reflect the intended population. By doing this, better estimates of the standard error are obtained. The Odds Ratio estimates, Standard Errors, 95% CI, and FDRs were provided to demonstrate the association between the individual factors and periodontitis. These regressions were repeated by smoking status to examine potential associations within smoking categories.

Random forests (RF) and classification and regression trees (CART) were employed to investigate associations and potential interactions between environmental factors, demographic and socioeconomic characteristics, and periodontitis disease status for each smoking status^76^. Specifically, for each smoking status RF was used to identify important factors (main effects and interactions) and then a single CART was used to visually investigate these relationships. Variables which were in the top ten for variable importance, were subsequently used to build a CART model with minimum node size of 100 and Bonferroni test for the stopping criteria. These methods were selected because the data involved many potentially correlated environmental factors and had the ability to allow nonlinearities and interactions without modeling them explicitly^77^. These analyses were performed using the “party”website (Version 1.0–25) package in R (Version 3.1.2). Repository for the data, R code, and SAS code can be accessed at https://github.com/joshuawlambert/PinarEtal2018.

## Supplementary information

Supporting Data
