# Supplementary material for: Epidemiologic evaluation of Nhanes for environmental Factors and periodontal disease
Source: Sci Rep. 2019 Jun 3;9:8227. doi: 10.1038/s41598-019-44445-3 (PMC6547714; doi:10.1038/s41598-019-44445-3)
Supplement: Supplementary file 1 — Supporting Data [file 41598_2019_44445_MOESM1_ESM.docx]

**Epidemiologic Evaluation of NHANES for Environmental Factors and Periodontal Disease**

P. Emecen-Huja^1^ ⃰ , H-F. Li^2^, J.L. Ebersole^3^, J. Lambert^4^, and H. Bush^5^

^1^Division of Periodontics, College of Dental Medicine, Medical University of South Carolina

Email: [emecenh@musc.edu](mailto:emecenh@musc.edu)

^2^Providence St. Joseph Health of Oregon, Medical Data and Research Center, Portland, OR

^3^University of Nevada, Las Vegas, School of Dental Medicine, Las Vegas, Nevada

^4^College of Nursing, University of Cincinnati, Cincinnati, OH

^5^Department of Biostatistics, College of Public Health, University of Kentucky, Lexington, KY

**Supporting Data Files for SREP-18-33747**

- Full descriptions of the sample design for these NHANES datasets are publically available at <https://www.cdc.gov/nchs/nhanes/>
- NHANES 1999-2004 datasets can be found at <https://wwwn.cdc.gov/nchs/nhanes/Default.aspx>
- Our data have been merged and processed and can be found at <https://github.com/joshuawlambert/PinarEtal2018/raw/master/data.zip>
- A unique identifier, SEQN <https://wwwn.cdc.gov/Nchs/Nhanes/1999-2000/DEMO.htm#SEQN> for the NHANES participant from our years of study (1999-2004) is included in these data.
- Survey-weighted logistic regressions were performed for each of the processed environmental factors, adjusting for age, gender, ethnicity, socio-economic status, smoking status and number of teeth. The R package “survey” was used in R (Version 3.1.2) for the survey-weighted logistic regression. Weights were constructed in SAS (Version 9.4) using a 6 year weighting design from the NHANES variable WTMEC2YR73 <http://www.cdc.gov/nchs/tutorials/Nhanes/SurveyDesign/Weighting/Task2.htm>
- Repository for the data, R code, and SAS code can be accessed at <https://github.com/joshuawlambert/PinarEtal2018>
